# Almond By-Products: A Comprehensive Review of Composition, Bioactivities, and Influencing Factors

**DOI:** 10.3390/foods14061042

**Published:** 2025-03-19

**Authors:** Vânia Silva, Ivo Oliveira, José Alberto Pereira, Berta Gonçalves

**Affiliations:** 1 Center for the Research and Technology of Agroenvironmental and Biological Sciences, CITAB, Inov4Agro, Universidade de Trás-os-Montes e Alto Douro, UTAD, Quinta de Prados, 5000-801 Vila Real, Portugal; al75372@alunos.utad.pt (V.S.); bertag@utad.pt (B.G.); 2 Centro de Investigação de Montanha, CIMO, LA SusTEC, Instituto Politécnico de Bragança, Campus de Santa Apolónia, 5300-253 Bragança, Portugal; jpereira@ipb.pt

**Keywords:** almond by-products, bioaccessibility, bioactive compounds, circular economy, sustainability

## Abstract

One of today’s major environmental and economic challenges is the fight against both agro- and industrial-waste. Almond production and industrial processing exemplifies this issue, as it generates tons of waste and by-products, with hulls and shells accounting for about 70% of the total fruit’s weight while skins represent about 6% of the shelled kernel. Since the edible kernel, about 23% of the total fruit weight, holds the highest commercial value, there has been growing interest within the scientific community in exploring the potential of these by-products. However, almond by-products contain a wide range of phytochemicals, mainly phenolic compounds (flavonoids and non-flavonoids), and triterpenoids, with great potential as antioxidant, antimicrobial, anti-inflammatory, and prebiotic properties. Although these by-products are being explored as alternative sources in the textile, pharmaceutical/cosmetic, and food industries, their primary use remains in livestock feed or bedding, or as biofuel. This review compiles recent scientific data on almond by-products’ phytochemical composition and bioactivities aiming to support sustainable and holistic agricultural practices.

## 1. Introduction

Today’s society is increasingly aware of sustainable development and healthy lifestyles in harmony with nature. The United Nations aims to achieve sustainable management and efficient use of natural resources by 2030, emphasizing the need to reduce food waste during production, transportation, and post-harvest handling through prevention, reduction, recycling, and reuse [1].

Agricultural production and related food industries generate high amounts of waste and by-products with significant impact on environmental, economic, and social sectors [2,3,4]. According to the United Nations Environment Programme (UNEP), around 17% (931 million tons) of global food production is wasted annually [5]. In Portugal, food waste per capita in 2022 was approximately 185 kg, with households being the main contributors [6]. Adopting sustainable and innovative strategies is essential to addressing this issue. By repurposing by-products and enhancing their value, eco-friendly practices can be promoted, ensuring a more sustainable production cycle [7]. This aligns with the key principles of the circular economy, which emphasize waste reduction, resource efficiency, and value creation. These by-products contain organic matter, minerals, and bioactive compounds with significant biological activity. Their effective use depends on thorough characterization, availability, and appropriate technologies for integration into new value chains. Effective use of these resources can contribute to sustainable practices, reduce waste, and create new opportunities for economic and environmental benefits [8].

The almond [*Prunus dulcis* (Mill.) D.A. Webb] is one of the nut trees with great economic relevance in Portugal and worldwide, valued for its nutritious edible kernels [9,10] and wide culinary applicability in bakeries and confectioneries [11,12,13]. Almond kernels are rich in proteins, low sugar content, unsaturated fatty acids, essential vitamins (especially α-tocopherol), minerals such as potassium, phosphorus, dietary fiber, phytosterols, and amino acids such as arginine [14,15,16,17]. These qualities, together with their sensorial appeal [18,19,20], make almonds a popular ingredient in the food industry [4,21,22] and a source of bioactive compounds with antioxidant properties that contribute to health benefits, especially when consumed in moderation [23,24,25,26]. Global almond production is projected to increase by 13% to 1.6 million tons in 2024/25, with consumption rising by 6% to match production levels, and exports growing by 3% to 1.1 million tons, driven by demand from the EU, China, and India [27,28]. In the season 2023/24, the global almond production (shelled basis) was 1.45 million tons with the United States leading at 77% of global production (1.12 million tons) [28]. Portugal produced 69,510 tons in 2023 [29]. The escalation in almond production poses the challenge of better management of waste and by-products as these arise in substantial quantities during their production and industrial processing [9,30,31] and, if not properly managed, can lead to significant environmental and economic harm [31,32]. Increasing consumer demand for natural, health-enhancing products underscores the relevance of almond by-products (hulls, shells, skins, and blanching water) which contain bioactive substances, particularly phenolic compounds, as well as essential nutrients, dietary fibers, fatty acids, and sugars with potential benefits for managing lifestyle-related diseases since they exhibit antioxidant [33,34,35], antimicrobial, and nutraceutical properties [4,36,37,38,39,40,41], and have appealing sensory characteristics [4,20,42,43]. Thus, almond by-products can be processed to isolate polyphenols that may be utilized in functional foods or nutraceuticals, which are incorporated into dietary supplements or pharmaceutical formulations, helping to reduce oxidative stress and inflammation-related disorders [4,9,36]. Advances in extraction and processing technologies, such as green solvents and enzyme-assisted methods, have improved the recovery of these compounds. Furthermore, integrating these bioactives into food systems can enhance shelf stability and nutritional value. The use of almond by-products exemplifies circular economy practices by transforming waste into valuable resources aligning with global sustainability goals, contributing to reduced environmental footprints and creating economic value through innovative applications. Despite the growing recognition of almond by-products as valuable resources, the transition from waste to high-value applications requires deeper exploration of their biochemical composition and functional properties to uncover innovative solutions for pressing global health challenges. Furthermore, the full potential of almond by-products remains underexplored, particularly in terms of clinical validation, bioavailability, and understanding synergistic effects among their bioactive components. New comprehensive studies are essential to identify, quantify, and understand the bioactive compounds present in these by-products, as well as to optimize extraction techniques that maximize yield and retain bioactivity, especially given the global scale of almond production worldwide.

Therefore, this review aims to provide a comprehensive overview of almond by-products’ composition, bioactivities, and factors influencing their potential, summarizing the latest findings and highlighting the role of these by-products in sustainable and circular economy practices, paving the way for future advancements in this promising field.

## 2. Methodology

This review was conducted following the PRISMA 2020 guidelines to ensure transparency and reproducibility in the selection, analysis, and reporting of relevant studies.

### 2.1. Search Strategy and Data Sources

A comprehensive literature search was performed using three major scientific databases such as Google Scholar and ScienceDirect. The search focused on identifying peer-reviewed studies written in English that investigated the phytochemical composition, bioactivities, and potential applications of almond by-products. The search terms used in the advanced search and respective search equations were: “almond by-products” OR “almond hull” OR “almond shell” OR “almond skin” OR “almond blanching water” OR “almond by-products bioactive compounds” OR “almond by-products bioactivities” OR “almond by-products antioxidant properties” OR “almond by-products anti-inflammatory properties” OR “almond by-products prebiotic properties” OR “almond by-products antimicrobial activity” OR “almond by-products bioaccessibility” OR “almond by-products potential applications” for the search engines ScienceDirect and Google Scholar. Relevant studies cited in the bibliographic references of reviewed articles were also directly accessed in addition to database searches.

### 2.2. Eligibility Criteria

#### 2.2.1. Inclusion Criteria

Peer-reviewed scientific studies published in English, studies providing data on the bioactive composition and functional properties of almond by-products, and studies published between 2015 and July of 2024 were included. Studies prior to 2015, found in citations of read articles, were included if they contained relevant data not available in recent publications. The inclusion of statistical and regulatory data from sources such as FAO, UNEP, USDA, Statista, and Eurostat followed specific criteria and were chosen based on relevance, credibility, and recency, prioritizing institutional and international sources for their rigorous methodology. Information was retrieved from official reports and databases to ensure accuracy and avoid third-party reinterpretations.

#### 2.2.2. Exclusion Criteria

Unpublished studies, dissertations, theses, personal communications, encyclopedia, conference abstracts, case reports, conference info, correspondence, data articles, discussion, editorials, errata, mini reviews, news, practice guidelines, short communications, and studies in which the outcomes of interest were not measured or reported were considered ineligible.

### 2.3. Study Selection and Data Extraction

**Figure 1:**
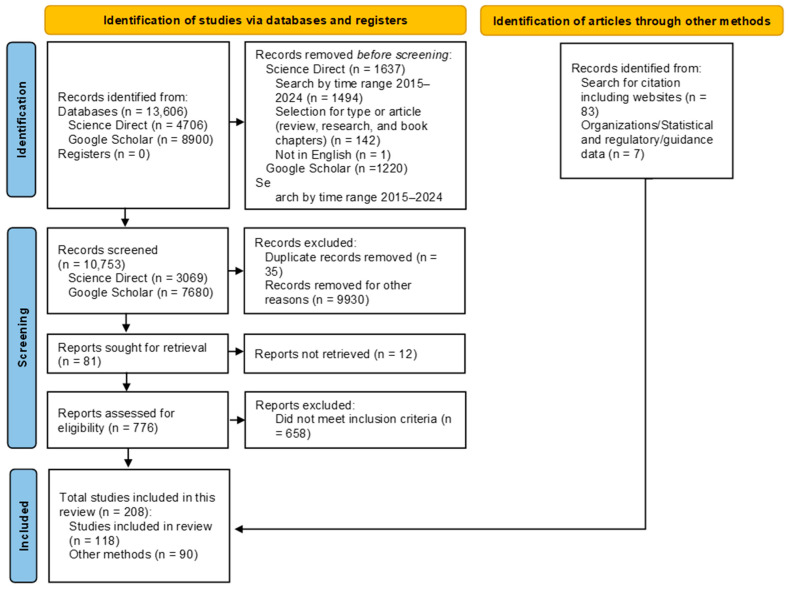
Flow diagram of the literature search and selection process, adapted from the PRISMA guidelines.

The search results were screened by reviewing titles and abstracts, and duplicates were removed through the Mendeley Library, as well as irrelevant studies. The full texts of eligible studies were then assessed based on their relevance to almond by-products’ composition, bioactivity, and potential applications. The extracted data were categorized according to the specific by-product analyzed (hull, shell, skin, blanching water) and the bioactivities studied. Particular emphasis was placed on reporting significant findings related to bioactive compounds, their properties, and factors influencing their bioavailability and functionality. The process of study selection and data extraction is summarized in the following flow diagram (Figure 1) which maps the number of records identified, included, and excluded, as well as the reasons for exclusions.

## 3. Almond By-Products

**Figure 2:**
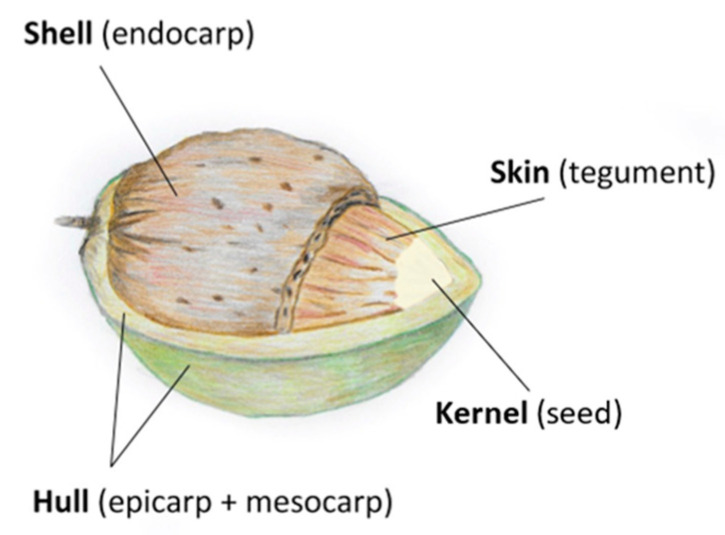
Almond morphology: structure and constituent parts.

During fruit development, the edible kernel is surrounded and protected by the epicarp and mesocarp (hull), endocarp (shell), and tegument (skin) [44] which are the constituent parts of the almond fruit (Figure 2). When the fruit’s maturation process has been reached (ripening stage), the hulls open and, once dried, and the fruit is ready to be harvested [9,13,44].

**Figure 3:**
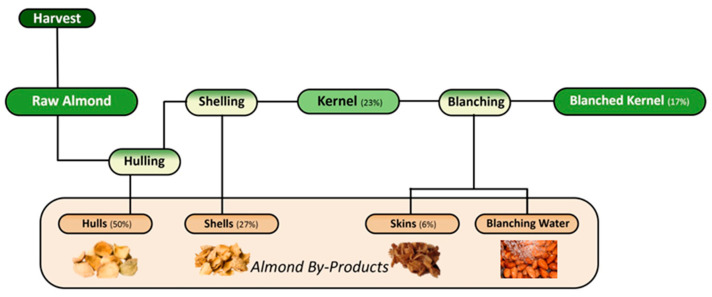
Almond industrial processing workflow to obtain the edible part (kernel) and resulting by-products, including the average proportion of each of its constituents.

The industrial processing to obtain the almond kernel consists of separating the external parts that surround it through sequential phases—namely hulling, shelling, and blanching—resulting in so-called almond by-products, namely hulls (AHs), shells (ASs), skins (ASks), and blanching water (ABW) [4,38,45] (Figure 3). Several studies indicate that the proportion of each almond constituent is highly dependent on the cultivar and corresponds to approximately 40–60% hull, 20–33% shell, and 15–31% kernel with skin of the total fruit weight. Additionally, the removed skin is the least representative constituent corresponding to only about 4–8% of the total kernel weight (Figure 3) [4,9,45,46]. Considering that the kernel is the edible and most valued/commercialized part, these values reflect that more than 70% of the almond fruit is by-product or waste material. Furthermore, the processes of hulling, shelling, and pelling are costly as they include the use of industrial machines, and the returns generated do not offset the investment in processing, thus threatening the sustainability and competitiveness of this industrial activity [47]. Furthermore, since these by-products are usually unvalued and discarded, or traditionally burned [48,49], while their hulls and shells, in particular, are primarily utilized as animal feed and bedding, their valorization becomes extremely important [9,13,44].

Finding sustainable uses for almond by-products can help reduce waste and environmental impact in the almond industry and maximize their contribution to sustainability efforts through zero waste generation [44]. Further exploration of their physicochemical features is essential to fully utilize almond by-products. This will help determine sustainable and competitive exploitation alternatives that align with current commercial practices [9].

### 3.1. Physical Characterization of Almond By-Products

Almond hull represents the most significant portion of the total fruit weight. AH becomes dry—with an average of 8–20% moisture content—leathery, and astringency as it matures, therefore it is not intended for nutritional purposes [50]. Consequently, AH has variable ripening stages and can range from being thin and dry (green-gray color), contributing minimally to the overall fruit, to being thick and fleshy (greenish color), making up a substantial portion of the fruit’s weight. The characteristics of AH significantly impact the ease of fruit removal from the tree, the drying process post-harvest, and the efficiency of hull removal [51]. An almond shell can vary in shape and size, with differences in appearance such as wrinkles and pores, and its hardness can also be highly variable [44]. AS is composed of two laminae, the outer one, rich in streaks and pores, and the inner one thinner, more compact and smoother [52]. The hardness of AS varies depending on the cultivar and depending on whether it is a soft shell, semi-hard shell, or hard shell [44,53], being linked to the total lignin content developed during nut growth [51], along with its morphology, fiber content, and the outer shell adherence [44,53]. The lignin complex provides structural support and rigidity to the shell cell walls of the shell and acts as an important kernel protection barrier against pests and pathogens [54,55].

Almond skin covers the kernel, serving as a protective barrier against oxidation and microbial contamination [50]. Depending on the cultivar, the skin may be thicker or thinner, smoother or more wrinkled [44]. The relevance of blanching water as a by-product has only recently gained attention, and research on its composition and potential uses is still in its early stages.

### 3.2. Chemical Characterization and Nutritional Content of Almond By-Products

#### 3.2.1. Almond Hull (AH)

Almond hull (AH) is rich in organic matter [56]. Reported variations in AH’s composition (Table 1) include sugar content (15.90–34.30%) [44,57,58,59,60], protein content (1.60–26.50%) [44,57,59,60,61], crude fiber (10.40–35.77%) [44,57,59,60,61], acid-detergent fiber (12.60–34.60%) [56,57,58,59,62], neutral detergent fiber (18.00–61.98%) [56,57,58,59], cellulose (6.60–20.70%) [44,63,64,65,66], and hemicellulose (6.00–12.86%) [44,65,66,67]. Ash content can range from 1.70 to 12.83% [44,57,59,60,61,66,68], exceeding 9% depending on the harvest method, in which case they are classified as “almond hulls and dirt” [9]. Almond hull is also rich in lignin (5.00–24.80%) [44,56,57,59,66], high in energy content [56], and high in organic matter (86.87–93.90%) [56,58,66].

#### 3.2.2. Almond Shell (AS)

As shown in Table 2, AS consists of approximately 51.80–62.00% crude fiber and around 90.10% neutral detergent fiber (NDF) [59]. AS is a highly fibrous and lignified material, primarily composed by cellulose (22.80–40.50%) [44,74,75,76,77,78], hemicellulose (19.70–35.20%) [44,52,74,75,76,77,78], and lignin (20.10–32.70%) [44,59,74,76,77,79]. Furthermore, AS also contain polysaccharides (56.10%) [79], ashes (0.55–8.70%) [76,77,78,79,80], and minerals—mostly potassium (4.30–12.30 g/kg) [44,59,78,79]—with substantial amounts of iron (0.04–1.64 g/kg) [59,78,79] and calcium (1.18–1.80 g/kg) [44,59,78,79], high carbon (45.60–50.50%) [74,81,82,83] and oxygen composition (37.97–45.94%) [74,81,82,83,84], and also high volatile matter (73.00–81.20%) [30,78,81,82].

#### 3.2.3. Almond Skin (ASk)

Almond Skin’s composition (Table 3) includes total dietary fiber (45.10–60.25%) [87,88,89,90,91] and soluble dietary fiber (2.70–3.80%) [87,88]. Besides fibers, ASk’s composition also includes fat (9.50–24.20%) [87,88,89], protein (10.30–12.80%) [87,88,90,91], and sugars (4.14–5.65%) [90,91]. Mohammed et al. [92] demonstrated that 1 g of ASk contains some minerals, mostly manganese (2.08%), zinc (2.96%), and iron (3.72%), and are rich in fatty acids, mainly oleic (43.08–56.00%) and linoleic (33.60–36.98%) [88,91,92].

## 4. Bioactive Compounds in Almond By-Products and Affecting Factors

### 4.1. Overview of Bioactive Compounds

Bioactive compounds are naturally occurring phytochemicals that include polyphenols, carotenoids, alkaloids, terpenoids, phytosterols, sulfur-containing compounds, and dietary fibers, among others, which contribute to food’s sensory qualities and serve as rich sources of natural antioxidants [93]. Among them, phenolic compounds (PCs) are particularly notable for their extensive biological and health-promoting properties [94,95,96,97] such as antioxidant [93,98,99,100], anti-inflammatory [98,100,101,102], antimicrobial [103,104,105], cytotoxic activity [98], and food additive/preservative [106,107,108]. Furthermore, PCs play an important role in plant physiology such as pigmentation, growth, reproduction, and resistance to pathogens [103].

### 4.2. Bioactive Compounds from Almond By-Products

Many studies have assessed the antioxidant capacity of bioactive compounds, particularly total polyphenols using varied methodologies such as 2,2-Diphenyl-1-picrylhydrazyl (DPPH), ferric ion-reducing antioxidant power (FRAP), 2,2′-Azinobis-(3-Ethylbenzothiazoline-6-Sulfonic Acid Assay) (ABTS), or Oxygen Radical Absorbance Capacity (ORAC) methods. Effectively, a multi-method approach offers a more complete antioxidant profile of these by-products. However, precise phenolic profiling requires advanced techniques like High-Performance Liquid Chromatography (HPLC) and Mass Spectrometry (MS), which enable detailed identification and quantification of individual phenolic compounds. By combining antioxidant assays with these analytical methods, researchers gain a comprehensive understanding of bioactive potential in almond by-products [9].

**Figure 4:**
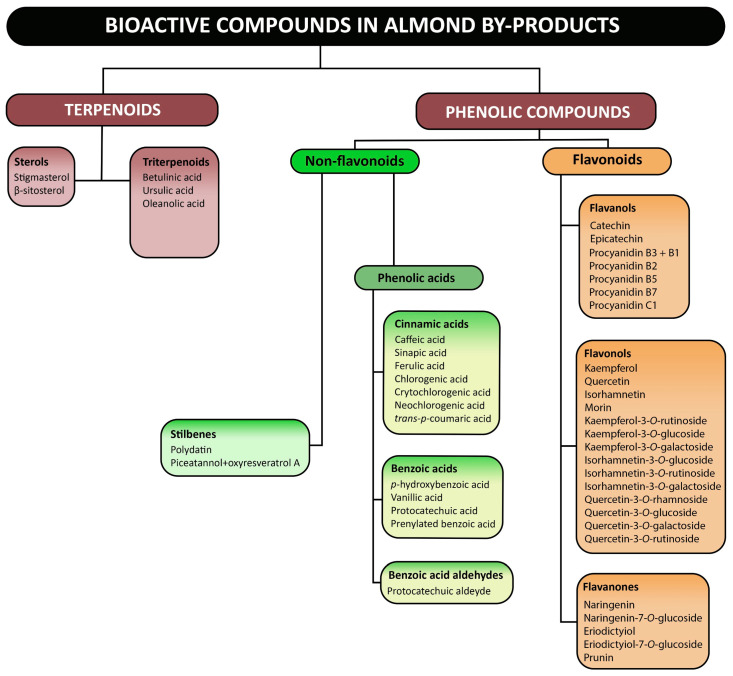
Main bioactive compounds found in almond by-products.

Thus, and through these methodologies, several authors identified the class of flavonoids and non-flavonoids as the main bioactive compounds present in almond by-products. In addition to these, terpenoids (sterols and triterpenoids) [36,109,110] have also been reported (Figure 4). The main subclasses of flavonoids found in almond by-products include: flavanols (monomers: catechins and epicatechins [36,39,88,111,112,113,114,115,116], and oligomers: procyanidins) [114,115], flavonols (e.g., kaempferol, quercetin, isorhamnetin, morin) [34,87,91,114,115,116,117], flavanones (e.g., naringenin, eriodyctiol, prunin) [87,114,115,116,118], and flavanonols (e.g., taxifolin) [39,114,115]. Additionally, the mainly subclasses of phenolic acids are: hydroxybenzoic acids (*p*-hydroxybenzoic, protocatechuic, vanillic, prenylated benzoic acid) [65,91,109,114,115], hydroxycinnamic acids (caffeic, sinapic, ferulic, chlorogenic, crytochlorogenic, neochlorogenic, and *trans-p*-coumaric) [36,91,114,115,116], and less mentioned, but also reported, benzoic acid aldehydes (protocatechuic aldehyde) [114,115]. Furthermore, almond by-products also contain polysaccharides, fatty acids, protein content, and volatiles [11,17].

Reported levels of phenolics [36,61,79,91,119] and antioxidant capacity [32,36,61,79,92,119] still vary considerably across studies. These variations in bioactive composition are essentially due to the type of solvent (e.g., ethanol, methanol, acetone), the respective percentage *v*/*v* (e.g., 70%, 80%), and the extraction method/procedure (e.g., time, temperature, pH) used [119,120]. They are also attributed to differences in the types of phenolics detected, type of detection methods, units of measurement, and standards used for expressing concentrations [11]. In fact, extraction methods significantly influence almond by-products’ quality and effectiveness (yield, purity, and bioactivity). Various techniques have been developed, each with their advantages and limitations. Traditional solvent extraction is the most common technique, using solvents like ethanol, but it can have environmental drawbacks. Newer methods like ultrasound-assisted extraction (UAE) and microwave-assisted extraction (MAE) offer faster, more efficient alternatives, using less solvent and preserving bioactive compounds. Enzymatic extraction is an eco-friendlier option, utilizing enzymes to break down cell walls, though it can be costly. Advanced methods like supercritical fluid extraction (SFE) and pressurized liquid extraction (PLE) offer high selectivity and efficiency but are more complex and expensive [17].

The content of total phenolics, flavonoids, *ortho*-diphenols, condensed tannins, and antioxidant capacity found in the literature on almond by-products are presented in Table 4.

In addition to this general bioactive composition, it is important to explore the phenolic profile of each by-product individually, as we will do below.

#### 4.2.1. Factors Affecting Bioactive Compounds in Almond Hulls

AH’s composition, bioactive content, and aspects like thickness and weight, vary depending on cultivation practices, almond cultivar, harvest conditions, processing, and environmental stress conditions or pest infections which can enhance flavonoid’s production as a protective response [50,59]. AH is a good source of bioactive substances, including phenolic compounds, mainly flavonol glycosides, hydroxycinnamic acids (e.g., chlorogenic acid), catechin, and protocatechuic acid [36,66,121,129], dietary fibers [4], triterpenoids (betulinic, ursolic, oleanolic acids) [109,130], and lactones [118], all contributing to its notable antioxidant capacity [26,66,121,129]. Studies highlight the diversity in AH phenolic profiles across cultivars and the influence of extraction methods.

Sang et al. [109] identified a novel prenylated benzoic acid derivative (3-prenyl-4-*O*-β-d-glucopyranosyloxy-4-hydroxylbenzoic acid) alongside catechin, protocatechuic acid, and ursolic acid [109], while Takeoka and Dao [110,130] reported triterpenoids, chlorogenic acid and their isomers, and sterols (stigmasterol and β-sitosterol) in the Nonpareil cultivar, with antioxidant activity even surpassing α-tocopherol in some tests [110,130]. Rubilar et al. [131] demonstrated that while AH and grape pomace share similar phenolic profiles, grape pomace exhibits superior antioxidant capacity, likely due to its higher flavonol content. In contrast, AH primarily contains hydroxybenzoic and cinnamic acid derivatives, with smaller amounts of flavan-3-ols, epicatechin, and glycosylated flavonols. This highlights the variability in antioxidant efficacy among agricultural residues, driven by differences in their phenolic composition [131]. Barreira et al. [122] noted a strong correlation between phenolic content and antioxidant strength among cultivars [122]. Kahlaoui et al. [121] identified chlorogenic acid, catechin, and protocatechuic acid as the dominant phenolics in seven cultivars, with Ultrasound-Assisted Extraction (UAE) yielding more diverse phenolic profiles like quercetin-3-glucoside, *p*-coumaric acid, epicatechin, and caffeic acid compared to conventional solvent extraction (CSE) [121]. Seasonal and irrigation variations also significantly impact the phenolic synthesis of AH. Functional assays have demonstrated the strong antioxidant and antimicrobial properties of AH, including effectiveness against multidrug-resistant bacteria like *Pseudomonas aeruginosa* and *Listeria monocytogenes*. Key compounds, such as naringenin-7-*O*-glucoside and isorhamnetin-3-*O*-rutinoside, are likely major contributors to this bioactivity [32].

Collectively, these studies emphasize AH’s potential as a source of bioactive compounds for nutraceutical and antimicrobial applications [32].

#### 4.2.2. Factors Affecting Bioactive Compounds in Almond Shell

Almond shell (AS), though low in nutritional value, contains trace amounts of bioactive compounds, such as phenols [119,123], flavonoids, and tannins, with moderate antioxidant capacity [79,119]. Interesting compounds include triterpenoids (betulinic [79], urosolic, and oleanolic acids), cinnamic acids (e.g., caftaric and chlorogenic acids), flavonols (e.g., kaempferol, isorhamnetin, and quercetin), flavan-3-ols (catechin and epicatechin), and flavanones (e.g., naringenin) [40,52,119]. Depolymerized lignin fractions, produced through mild acid hydrolysis, have shown antioxidant potential [125]. Additionally, *O*-acetylated xylo-oligosaccharides (DXO) and their de-acetylated form (DeXO) extracted from almond shells demonstrate immunostimulatory potential, exhibiting mitogenic activity and enhancing T-cell proliferation in rat thymocytes [132]. Valdés et al. [119] demonstrated that microwave-assisted extraction (MAE) with response surface methodology (RSM) have optimized the recovery of antioxidants from AS, with temperature and pH influencing phenolic content and antioxidant efficacy. Key compounds like chlorogenic acid and catechin, rich in hydroxyl groups, significantly contribute to the strong antioxidant potential of AS [119]. These findings highlight the AS’s potential as a source of bioactive compounds with antioxidant and immunomodulatory benefits.

#### 4.2.3. Factors Affecting Bioactive Compounds in Almond Skin

Almond skin (ASk) is the most studied almond by-product, and despite its relatively low market value, it is rich in bioactive compounds, particularly phenolics which represent more than 60% of the total phenolic content of the almond nut [11,91,133,134,135]. Key phenolics include flavonoids in their aglycone and glycosides forms—like quercetin, kaempferol, naringenin, isorhamnetin, catechin, and epicatechin—as well as phenolic acids like chlorogenic, protocatechuic, *p*-hydroxybenzoic, vanillic, caffeic, ferulic, and *p*-coumaric acids, which are associated with high radical scavenging activity and protective health benefits [9,50,88,89,115,136]. The concentration of these bioactive compounds varies by cultivar and extraction methods [39,137]. Industrial thermal treatments like blanching and roasting affect polyphenol stability, leading to the degradation and loss of bioactive compounds [138]. Blanching ASk (~100 °C) can result in notable polyphenol degradation [127], while roasting (up to 200 °C) generally preserves more phenolics [138] and enhances antioxidant activity [133] through increased extraction efficiency and the formation of new antioxidants via Maillard reactions, caramelization, and thermo-oxidation [139,140], with maximum benefits observed at 200 °C for 20 min [141]. Advanced extraction techniques, like Ultrasound-Assisted Extraction (UAE), are highly efficient, enabling the recovery of up to 87% of total polyphenols as procyanidins [142]. ASk is rich in phenolic compounds, particularly flavonoids, with around 95% of the almond’s flavonoids concentrated there, primarily as isorhamnetin-3-*O*-rutinoside and isorhamnetin-3-*O*-glucoside which together make up over 70% of the identified flavonoids [33]. Flavanols and flavonol glycosides are the most abundant phenolic compounds in ASks, representing up to 38–57% and 14–35% of the total phenolics, respectively [114]. Furthermore, flavonoids in their aglycone forms exhibit higher efficiency in terms of radical scavenging activity compared to their glycoside forms [34].

Sang et al. [143] identified nine phenolic compounds from the ethyl acetate and n-butanol fractions of almond skins, namely 3′-*O*-methylquercetin 3-*O*-β_-D_-glucopyranoside, 3′-*O*-methylquercetin 3-*O*-β_-D_-galactopyranoside, 3′-*O*-methylquercetin 3-*O*-α-_L_-rhamnopyranosyl-(1→6)-β-_D_-glucopyranoside, kaempferol 3-*O*-α-_L_-rhamnopyranosyl-(1→6)-β-_D_-glucopyranoside, naringenin 7-*O*-β-_D_-glucopyranoside, catechin, protocatechuic acid, vanillic acid, and *p*-hydroxybenzoic acid. Catechin and protocatechuic acid showed very strong DPPH radical scavenging activity, while the other compounds, except kaempferol, exhibited strong antioxidant activity [143]. Frison-Norrie and Sporns [144,145] quantified four flavonol glycosides (Kaempferol rutinoside and glucoside, and isorhamnetin rutinoside and glucoside) [144,145], with isorhamnetin rutinoside being the most abundant across the 16 cultivars [144]. Amarowicz et al. [146] identified procyanidins B_2_ and B_3_ as dominant compounds beyond hydroxicinamic acids (*p*-cumaric, ferrulic, vanilic, and caffeic acids), isorhamnetin, quercitin, kaempferol, delphinidin, and cyanidin [146]. Complementing this, Arráez-Román et al. [112] compared CE and HPLC coupled with ESI-TOF-MS for phenolic profiling, finding HPLC to be more efficient and identifying 23 coumpounds (phenolic acids and flavonoids) in just 9 min [112]. Research by Bolling et al. [111], using reverse-phase HPLC coupled with negative-mode ESI-MS, quantified 16 flavonoids and 2 phenolic acids in ASk, showing that hot water blanching yielded the highest polyphenol recovery, while solvent-assisted extraction on liquid nitrogen not only increased aglycone recovery but decreased flavonol glycosides [111].

Garrido et al. [127] identified 31 phenolic compounds, mainly flavan-3-ols and flavonol glycosides, but also hydroxybenzoic acids and aldehydes, flavonol aglycones, flavanone glycosides, flavanone aglycones, and dihydroflavonol aglycones. Research reported that roasting ASk significantly increased its antioxidant capacity (ORAC values) and phenolic content, outperforming blanched samples [127]. Additionally, Mandalari et al. [88] found that natural ASk had higher total phenolic content and antioxidant capacity (DPPH method) compared to blanched skin. The study showed that while blanching significantly reduces polyphenol content, the blanched skin still retains bioactive compounds with antioxidant properties. Identified compounds in both ASks were flavonols, flavanols, hydroxybenzoic acids, and flavanones, with catechin, epicatechin, kaempferol, and isorhamnetin-3-*O*-rutinoside being the most common flavonoids [88]. Similarly, Smeriglio et al. [116] confirmed that blanching reduced polyphenol content by over 60%, while natural skin exhibited the highest antioxidant, antimicrobial (particularly against Gram-negative bacteria), and cytoprotective properties. The 21 derivatives of phenolic compounds identified (by RP-HPLC-DAD) included flavanones, flavonols, flavan-3-ols, and phenolic acids, with naringenin being the most abundant, followed by kaempferol-3-*O*-rutinoside, kaempferol-3-*O*-glucoside, kaempferol, and eriodictyol-7-*O*-glucoside [116]. Contrastingly, Ingegneri et al. [91] found that blanched ASk had higher phenolic and antioxidant levels than blanching water, with isorhamnetin-3-*O*-glucoside being the most abundant flavonoid in both by-products. Blanched skin samples demonstrated antiviral activity against herpes simplex virus 1 along with high fiber (≥52.67%) and protein (≥10.99%), as well as low fat (≤15.35%) and sugars (≤5.55%), showcasing their potential to be nutritionally valuable [91]. Contrary to observations by other authors, Bolling et al. [126] noted that while pasteurization did not significantly impact phenol or antioxidant levels [126], roasting (146 °C for 14 min) reduced total phenols and antioxidant activity, although phenolic acids remained stable. They also noted that long term storage significantly enhanced phenolic content and antioxidant potential by up to 200%. This suggests that controlled storage can increase bioactive compound availability and potentially boost almonds’ health benefits over time [126]. Furthermore, a study on California almond varieties (Nonpareil, Butte, and Carmel) identified polydatin in ASk extracts using UHPLC-MS, further showcasing the phenolic diversity of ASk [147].

#### 4.2.4. Factors Affecting Bioactive Compounds in Almond Blanching Water

Blanching almonds leads to a significant leaching of polyphenols from the skin into almond blanching water (ABW), enriching it with valuable bioactive compounds [33,88,127]. Key phenolic compounds in ABW include naringenin-7-*O*-glucoside, kaempferol-7-*O*-rutinoside, catechin [33], and various phenolic acids (protocatechuic, chlorogenic, coumaric, *p*-hydroxybenzoic, and vanillic acids). Flavonoids like eriodyctiol, naringenin, quercetin, kaempferol, catechin, and epicatechin [39,128,148] also contribute to its antioxidant properties by helping in neutralizing free radicals and reducing oxidative stress [136]. However, compounds like kaempferol, quercetin, isorhamnetin, quercitin-3-*O*-galactoside, and quercitin-3-*O*-rutinoside which are less water-soluble, have limited transfer to ABW remaining in the ASk [33,88,117]. Hughey et al. [117] demonstrated that blanching (100 °C within 10 min) significantly increased phenolic leaching (~90%) to hot water, with first-order kinetics observed for compounds like catechin and epicatechin, resulting from the degradation of polymeric procyanidins [117]. Additionally, ABW contains polydatin (6.33–8.43 μg/100 g) and low concentrations of piceatannol + oxyresveratrol (0.91–2.55 μg/100 g) [147], which have potential anti-inflammatory effects helping to mitigate inflammation-related conditions [148] and providing opportunities for its use as a natural ingredient in food, cosmetics, and nutraceuticals [91,149]. Although almond blanching water is generally safe for consumption, proper processing and handling procedures should be followed to ensure food safety and quality [91].

Table S1 (Supplementary Materials) presents the main individual phenolic compounds and terpenoids, along with their respective contents, as reported in the literature for almond hulls, shells, skins, and blanching water.

## 5. Biological Activities of Almond By-Products and Influencing Factors

As mentioned, almond by-products are rich in bioactive compounds, with notable biological activities and significant potential for various health-benefits such as: antioxidant activity/antiradical [122,150,151], antimicrobial [116,152], anti-inflammatory [153], antiproliferative [154], prebiotic [11,155], photoprotective [128,156,157], and antiviral properties [158]. The diverse biological activities of phenolic compounds have been reported in several studies, including both in vitro and in vivo, in animals and humans [9,158,159,160]. Almond skins, for example, contain high levels of flavonoids like catechin and epicatechin, which help reduce oxidative stress and support skin health. The beneficial effects of ASk are enhanced when ASk polyphenols interact with vitamins C and E, boosting antioxidant defenses and reducing LDL oxidation [11,150]. Additionally, AH contains triterpenoids like betulinic acid, oleanolic acid, and ursolic acid [130], which are known for several health benefits such as anti-inflammatory, anticarcinogenic, antiplasmodial, antiulcerogenic, analgesic, hepato- and cardio-protective, and antimicrobial and antiviral properties, as well as activity against the human immunodeficiency virus (HIV) [161]. ASk is also high in dietary fiber, particularly insoluble fiber, which aids digestion, may increase satiety [162,163], and contributes to prebiotic effects that support gut health by promoting beneficial bacteria [88,164]. The fiber content further enhances anti-inflammatory benefits, with potential impacts on reducing inflammation-related conditions [165]. Furthermore, ASk contributes to the characteristic color and flavor of almonds and almond-derived products, adding a slight bitterness and contributing to their sensory appeal [90,166]. Similarly, ABW, enriched with phenolic acids and flavonoids due to polyphenol leaching during the blanching process, has antioxidant and anti-inflammatory potential. Together, these by-products offer valuable functional properties, positioning them as promising ingredients for food, cosmetic, and pharmaceutical applications, with benefits ranging from enhancing gut health to providing natural antioxidant protection.

### 5.1. Factors Affecting Antioxidant Capacity of Almond By-Products

Phenolic compounds present in almond by-products have been shown to exhibit various biological effects, particularly antioxidant activity. This activity is largely attributed to matrix redox properties, which are effective in neutralizing free radicals, quenching singlet and triplet oxygen, and breaking down peroxides [167]. Researchers have evaluated the antioxidant activity of these compounds using different methods such as DPPH [88,143,151,168], FRAP [116,126,168], ABTS [116], and ORAC [114,116,127,151,168]. These methods, with different mechanisms of action, complement each other in providing a comprehensive evaluation of antioxidant capacity. The DPPH method measures the ability of antioxidants to scavenge DPPH radicals, while FRAP and ABTS methods assess the reduction potential of antioxidants and can evaluate molecules with varying polarity and reduction power [169]. The ORAC method quantifies the ability of antioxidants to neutralize reactive oxygen species (ROS) by monitoring the loss of fluorescence from a probe, which is diminished when ROS are present [170].

Valdés et al. [113] observed high DPPH scavenging activity in ASk extracts from seven cultivars (Spanish and American), reaching up to 90%, with post-drying processes enhancing antioxidant capacity [113]. Maximum total phenolics and antioxidant capacity were achieved with hydroethanolic extracts (70% *v*/*v*) which was in accordance with other studies [119,121]. Similarly, Bottone et al. [137,171] highlighted the superior performance of hydroethanolic extracts compared to ethanol-only extracts, especially in the skin [137,171] and hull [121] of specific cultivars like ‘Pizzuta’ [121,137] and ‘Fascionello’ [137]. Additionally, Siriwardhana and Shahidi [124] found almond skins and hulls to exhibit up to 13 and 10 times greater, respectively, than whole seeds, demonstrating remarkable free radical scavenging and hydrogen peroxide-reduction capabilities. Notably, 100% DPPH scavenging activity was observed for skin (at 100 ppm) and hull (at 200 ppm) extracts [124]. Additionally, Wijeratne et al. [34] reported that defatted skin and hull extracts were 9–10 times richer in phenolics than whole seeds, with extracts (at 50 ppm) effectively inhibiting human low-density lipoprotein (LDL) oxidation (mainly skin extracts) and DNA damage (mainly hull extracts), showing excellent metal ion chelation abilities. Furthermore, key flavonoids like quercetin and kaempferol derivatives were identified by HPLC in all extracts [34]. Genotypic and geographic variations also influence antioxidant capacities. Sfahlan et al. [123] further explore the variability in antioxidant activity across different genotypes, highlighting the value of selecting specific genotypes for high phenolic content, with hulls generally outperforming shells [123]. The ethyl acetate-soluble fraction from almond shell hydrolysis showed DPPH scavenging ability and fish oil preservation benefits, with phenolic-rich hydrolyzed extracts providing activity comparable to synthetic antioxidants like propyl gallate [125]. Specific polyphenols, such as chlorogenic acid in hulls [109,123,124] and isorhamnetin in skins [33,91,144], are key contributors to antioxidant activity and their associated health benefits. Hull extracts have shown strong oxidation inhibition in oils and meat systems, attributed to phenolic acids like caffeic, ferulic, *p*-coumaric, and sinapic acids [35]. ASk extracts enhance endogenous antioxidant defenses by inducing enzymes such as glutathione peroxidase (GPx), superoxide dismutase (SOD), and catalase (CAT) [165,172] while modulating oxidative stress biomarkers [165], including glutathione levels [173], and activating signaling pathways like nuclear factor-E2-related factor 2 (Nrf2) [165,172] and antioxidant response element (ARE)-reporter gene activity in vitro [172]. Additionally, polyphenol-enriched almond hull extracts exhibit strong antioxidant properties by scavenging reactive oxygen species (ROS) and regulating cellular redox balance in oxidative stress models such as Caco-2 cells [129]. Similarly, acetone extracts from almond hulls have been shown to protect human erythrocytes from oxidative damage and degradation of membrane proteins caused by hydrogen peroxide that may have resulted from the integration of antioxidants into cell membranes or translocation to the cytosol [173]. Human trials further demonstrate that almond consumption can mitigate oxidative DNA damage and lipid peroxidation in high-risk groups, such as smokers [174,175]. In vitro studies confirm the lipid peroxidation inhibition capacity of almond skin extracts [175,176]. Recently, tested aqueous extracts from almond skins of different cultivars were tested for the first time as a natural additive in meat burgers. The study confirmed water as an effective, economical, and eco-friendly solvent for extracting phenolic compounds, achieving levels close to 2.00 mg GAE/g of sample [177].

### 5.2. Factors Affecting Antimicrobial Effect of Almond By-Products

Research has demonstrated that polyphenols from almond by-products (mainly from the hull and skin) possess antimicrobial activity against human and foodborne pathogens by directly targeting microorganisms and inhibiting virulence factors [38,178]. Effectively, the antioxidant and antimicrobial power of almond skin extracts was attributed to the presence of polyphenols such as catechin, epicatechin, isorhamnetin, kaempferol, naringenin, and protocatechuic acid [32,152], as well as triterpenoids and hydroxycinnamic acids in the almond hull [36]. Additionally, polyphenols exhibit synergistic effects when combined with antibiotics, enhancing efficacy against resistant pathogens [179]. Some in vitro studies have demonstrated the antibacterial effect of almond by-products against Gram- and Gram+ bacteria, which is sometimes greater than commercial antibiotics such as Gentamicin. Extracts from skins proved to be effective against *Staphylococcus aureus*, *Enterococcus faecalis*, *Pseudomonas aeruginosa*, *Listeria monocytogenes*, *Salmonella enterica*, *Escherichia coli*, *Streptococcus mutans*, and *Serratia marcescens* [32,116,152,180]. Furthermore, skin extracts showed a potent inhibition of the proliferation of *Helicobacter pylori*, mainly due to the presence of protocatechuic acid [181]. Moreover, polyphenolic extracts from almond skin have antiviral properties, suppressing the production and spread of herpes simplex virus type 1 (HSV-1) infection [157], with flavonones identified as a key compound responsible for this inhibition [178]. Still, in this sense, results from Arena et al. [182] showed that natural almond skin extracts significantly reduced HSV-2 replication and promoted the production of both Th1-related cytokines (e.g., IFN-α, IL-12, TNF-α, IFN-γ) and Th2 cytokines (e.g., IL-4, IL-10), while blanched skin extracts showed limited influence on viral replication. These findings suggest that natural skins enhance PBMC immune responses against viral infections by activating both Th1 and Th2 pathways [182]. In parallel, almond hull extracts also demonstrated antimicrobial activity against *Escherichia coli*, *Staphylococcus aureus*, *Salmonella typhimurium*, *Pseudomonas aeruginosa*, *Enterococcus faecalis*, and *Listeria inocua* [32,36,183]. Lastly, D’Arcangelo et al. [184] demonstrated that almond hull extract (AHE), high in phenolic acids and flavonoids, possesses significant antimicrobial activity against planktonic cells of *Escherichia coli* and staphylococcal strains. Additionally, AHE exhibited notable antibiofilm effects, effectively inhibiting bacterial adhesion and promoting the removal of mature biofilms. Safety testing on human fibroblasts revealed that AHE is non-toxic to normal human cells, making it a promising candidate for antimicrobial applications [184].

### 5.3. Factors Affecting Anti-Inflammatory Effect of Almond By-Products

Almond by-products have been recognized for their anti-inflammatory properties that are attributed to their rich polyphenolic content like flavonoids, phenolic acids, and proanthocyanidins, UFAs, and protein hydrolysates which modulate inflammatory pathways by targeting oxidative stress and suppressing key inflammatory mediators. In fact, in vivo and in vitro studies have evidence of the potential anti-inflammatory effect of almond by-products [11,175]. Almond consumption, which includes bioactive components found in skins, has been linked to decreased inflammatory biomarkers, such as C-reactive protein (CRP), in both human and animal models, suggesting its potential role in dietary interventions to manage chronic inflammation [175]. In fact, acetonic extracts from ASk demonstrated significant potential for managing intestinal inflammation by effectively inhibiting TNF-α and reducing reactive oxygen species (ROS) release, even at low concentrations (5 μg/mL). This approach not only addresses solubility challenges but also enhances the extract’s bioactivity, making it a promising candidate for use in dietary supplements targeting inflammatory conditions [185]. Additionally, in vivo studies demonstrated that polyphenols from aqueous extracts of ASk improved epithelial barrier function in rodent models by restoring villin and MUC3 mucin levels in TNBS-induced colitis in rats [186]. Similarly, Mandalari et al. [153] demonstrated that natural almond skin (NS) powder significantly alleviated symptoms of dinitrobenzene sulfonic acid (DNBS)-induced colitis in mice. Oral administration of NS powder (30 mg/kg daily) effectively reduced inflammation markers, including NF-κB and *p*-JNK activation, TNF-α and IL-1β production, and neutrophil infiltration. Additionally, NS powder improved intestinal health by reducing diarrhea, body weight loss, and intestinal inflammation [153]. Furthermore, research on almond skin polyphenols (ASP) showed that consuming it (450 mg) in milk significantly increased plasma levels of catechin and naringenin in adults enhancing oxidative stress markers, with a 212% increase in the GSH/GSSG ratio and a 26–35% boost in GPx activity. Additionally, LDL’s resistance to oxidation improved by over 140% compared to milk consumption alone, further underscoring the bioavailability of ASP and their antioxidant potential [175]. Ethanolic extracts from green ASk showed renal protective effects in a rat model of ferric nitrilotriacetate (Fe-NTA)-induced renal cell carcinoma (RCC). Doses of 25, 50, and 100 mg/kg administered orally over 22 weeks mitigated RCC by reducing renal nodules, tissue discoloration, tumor-promoter markers, oxidative stress biomarkers in serum, and inflammatory markers such as IL-6, IL-1β, TNF-α, PGE2, and NF-κB in a dose-dependent manner. Histopathological analysis revealed reduced necrosis, normalized Bowman capsule size, and decreased inflammatory cell infiltration [187].

The anti-inflammatory effects of almond by-products, particularly skins, demonstrated in vitro and in animal studies, position them as valuable ingredients in functional foods, nutraceuticals, and even pharmaceutical formulations aimed at managing chronic inflammatory conditions, including cardiovascular diseases, metabolic syndrome, and arthritis. However, the efficacy of bioactive compounds in almond by-products depends on their bioavailability, which varies among individuals. Human data remain scarce, requiring cautious integration into dietary plans, and therefore almond by-products should be recommended as a complementary, not primary, anti-inflammatory strategy. Patients with inflammatory conditions may benefit from these products in combination with conventional therapies, but more research is needed. Since almond by-products may help reduce biomarkers of inflammation, as suggested by animal models, there should be an adaptation to specific conditions related to, for example, oxidative stress and chronic inflammation, such as metabolic syndrome or mild inflammatory disorders. Monitoring of food tolerability would be advisable. Gradual inclusion of almond by-products (e.g., powders or extracts) in functional foods to assess individual tolerability and ensure that there are no adverse interactions, especially in patients with nut allergies or gastrointestinal sensitivities. While by-products show potential, whole almonds (with skins) have the strongest evidence for reducing markers of inflammation in humans. Thus, whole almonds are recommended in combination with emerging by-product applications for a synergistic approach. Healthcare practitioners should be cautious in making definitive claims about health benefits without patient-specific data. Continued exploration of their bioactivity through both mechanistic and clinical studies will help solidify their role in health applications.

### 5.4. Factors Affecting Prebiotic Properties of Almond By-Products

Almond by-products, have shown significant potential as prebiotic agents due to their high content of dietary fibers and polyphenols [152], xylooligosaccharides (XOS), polysaccharides, and hemicellulose [188,189]. These compounds are resistant to digestive enzymes in the upper gastrointestinal tract and are metabolized by gut microbiota into bioactive metabolites [4,11,152,155]. Thus, selective promotion of the growth and activity of beneficial gut microbiota enhance gut barrier function, thereby contributing to improved gut health and systemic benefits while reducing pathogenic species [4,11,155,178,190]. Effectively, an in vitro digestion model revealed that dietary fibers from almond skin promoted the growth of beneficial bacteria, such as *Clostridium coccoides* and *Eubacterium rectale* [152], due to butyrate production, resulting in the intestinal microbiota’s metabolization of unsaturated fatty acids (UFAs) and polyphenols present in the skin during fecal fermentation [11,191,192]. In vivo human studies have demonstrated that the consumption of almond skins selectively increases the abundance of beneficial bacteria such as *Lactobacillus* and *Bifidobacterium* species in fecal samples suppressing the proliferation of *Clostridium perfringens* [155]. In vivo animal studies were also developed by mainly looking at how adding almond hull to animal feed affects ruminant performance and digestibility [193,194], highlighting the potential of almond by-products as functional ingredients in animal nutrition. Beyond ruminants, almond hulls have proven effective in other species. For example, using insoluble fiber from hulls in growing pigs’ diets improved growth rates, reduced ammonia emissions, and had no adverse effects on digestion or microbiota composition [195]. In poultry, including up to 2% almond hulls in broiler diets, enhanced growth performance, nutrient digestibility, and reduced microbial loads and noxious gas emissions, indicating their potential as a sustainable feed ingredient [196]. Similarly, prime almond hulls used as an energy and fiber source at levels of up to 6–9% showed no negative impact on body weight gain in broilers while increasing beneficial bacterial populations, such as the genus *Clostridium* and *Oscillospira* [60].

As already mentioned, polyphenols are bioaccessible in the upper gastrointestinal tract and can potentially be absorbed during the human digestive process. However, its bioaccessibility appears to be significantly affected by the type of food matrix used and the processing method [191,197]. In this sense, Liu et al. [198] concluded that the roasting process may slightly reduce prebiotic effects, despite significantly improving metabolic effects [199]. The prebiotic properties of almond by-products, particularly skins, make them valuable for functional food development and gut health supplements [11].

### 5.5. Other Biological Activities of Almond By-Products and Influencing Factors

Similar to the activities above, the bioactive compounds present in almond by-products also have anticancer potential, largely attributed to their antioxidant properties [178]. This is because oxidative stress, considered one of the basic processes involved in the initial stages of carcinogenesis, is effectively mitigated by these compounds, highlighting their role in cancer prevention [17]. Both in vitro and in vivo studies highlight their efficacy in inhibiting cancer cell proliferation, inducing apoptosis, and interfering with cancer progression pathways. For instance, acetonic extracts, essentially rich in flavonoids and phenolic acids, from almond skins demonstrated strong cytotoxicity against human breast cancer cell lines (MCF-7 and MDA-MB-468) [199] while terpenoids, mainly betulinic acid, extracted from almond hulls also exhibited high cytotoxicity against MCF-7, surpassing traditional chemotherapeutics like 5-fluorouracil [154,200]. In addition, UFAs (oleic and linoleic acids) present in almond skin oil have shown antiproliferative effects against colon carcinoma cells through pathways involving BMP-2 and β-catenin [201]. Similarly, polyphenol-enriched hydroacetonic extracts from almond hulls inhibited osteosarcoma (Saos-2) cell migration and induced mitochondrial dysfunction and caspase-mediated apoptosis outperforming some clinical anticancer agents [17,154,202]. Additionally, polysaccharide fractions—water-soluble (WSP), oxalate-soluble (OXSP), and hydrochloric acid-soluble (ASP)—extracted from almond hulls displayed strong cytotoxic effects against colon carcinoma (Caco-2) and melanoma (B-16) cell lines. Among these fractions, ASP showed the strongest antioxidant and antiproliferative activities, attributed to its high galacturonic acid content, low esterification rate, and average molecular weight [203]. Beyond anticancer properties, almond by-products exhibit protective effects against chronic diseases, such as cardiovascular diseases (CVD), dyslipidemia, diabetes, liver damage, and neurodegenerative conditions [172,175,178,188,204,205,206,207]. Regular almond consumption has been linked to improved lipid profiles, reduced LDL oxidation [178], and better glycemic control due to the synergistic actions of UFAs, fiber, and polyphenols [178,204]. Moreover, procyanidin-enriched almond skin extracts have shown hepatoprotective effects by reducing hepatic enzyme levels and enhancing antioxidant defenses [172]. Furthermore, almond skin and blanching water also display photoprotective effects, reducing UV-induced skin damage [128,156]. Research has demonstrated that almond skin extract can protect against oxidative stress [156] and that almond skin blanching water extract also reduces erythema (50.5%) caused by UV-B exposure [128]. These benefits have been validated in clinical trials, highlighting the broader health potential of almond-derived products.

In general, the studies present compelling evidence on the health benefits of almond by-products but also highlights the need for further research to address existing gaps and improve their application in clinical settings. A major issue is the lack of standardization in extraction methods and concentrations of bioactive compounds, making it difficult to compare results across studies. Additionally, while the bioactive effects of these by-products are well-documented in vitro, there is limited in vivo validation or robust clinical trial data to confirm their efficacy in humans. The molecular mechanisms underlying their effects, although partially explored, require deeper investigation to understand their full therapeutic potential. Another limitation is the minimal focus on toxicity and safety data like dosage, optimization, and long-term safety studies, which are essential for the development of these compounds into nutraceuticals or pharmaceuticals. Furthermore, the studies primarily examine isolated compounds, neglecting the potential synergistic effects of whole extracts. For all biological activities, improving bioavailability and developing effective delivery systems are critical to maximizing their therapeutic potential. Addressing these gaps through comprehensive research will enhance the applicability and reliability of almond by-products in health promotion and disease prevention.

The main biological activities of almond by products are mentioned in Table 5. It is important to note that while almond by-products exhibit the aforementioned biological activities, the extent and specific mechanisms of action can vary based on several factors including the specific bioactive compounds present.

## 6. Conclusions

The almond industry generates significant amounts of bio-waste, primarily in the form of hulls, shells, skins, and blanching water, which collectively represent a substantial portion of the whole almond fruit. Almond by-products represent a valuable resource with diverse bioactivities and health benefits. Hulls, skins, and blanching water are good sources of phenolic compounds, and other bioactive substances with notable antioxidant, antimicrobial, prebiotic, antitumor, antiviral, and photoprotective properties with promise in the pharmaceutical, food, and cosmetic industries. Key bioactive compounds in almond skin include flavonoids in both aglycone and glycoside forms—such as naringenin, eriodyctiol, quercetin, kaempferol, and isorhamnetin, naringenin-7-*O*-glucoside, eriodictyol-7-*O*-glucoside, and isorhamnetin-3-*O*-rutinoside—as well as catechin and epicatechin, hydroxybenzoic and hydroxycinnamic acids (e.g., protocatechuic, *p*-hydroxybenzoic, chlorogenic, vanillic, *trans-p*-coumaric, and caffeic acids), dietary fibers, and proanthocyanidins (particularly B2 and B3). Almond hulls contain hydroxycinnamic acids (chlorogenic, neochlorogenic, cryptochlorogenic), catechin, protocatechuic acid, fibers, and triterpenoids like betulinic, ursolic, and oleanolic acids. Almond shells are rich in cinnamic acid derivatives, kaempferol and quercetin glycosides, aglycones, catechin, epicatechin, naringenin and isorhamnetin derivatives, cellulose, fibers, and triterpenoids. Almond blanching water also contains phenolic acids (protocatechuic, *p*-hydroxybenzoic, vanillic, chlorogenic, and coumaric acids), catechin, epicatechin, naringenin, eriodyctiol, quercetin, and kaempferol. Optimizing processing and storage is crucial to enhance bioactive extraction. Developing new, sustainable extraction techniques using green strategies like Microwave-Assisted Extraction (MAE), Ultrasound-Assisted Extraction (UAE), and Supercritical Fluid Extraction (SFE), is essential for their efficient and eco-friendly purification.

Overall, harnessing the bioactive compounds in almond by-products offers a holistic approach to promoting human health, environmental sustainability, and economic viability in the food industry. Continued research and innovation in this area are essential for maximizing the potential of almond by-products and realizing their benefits for both consumers and producers.

## 7. Challenges and Future Perspectives

Almond by-products are widely generated, particularly in major almond-producing regions such as California. California alone produces around 1 million tons of shells and 3 million tons of hulls annually, alongside smaller quantities of almond skin and blanching water. Despite their abundance, these by-products are often underutilized or fetch low economic value. For instance, almond hulls typically sell for around $100 per ton, while shells add minimal value [208]. That is why it is so important to reuse these by-products to make them profitable.

However almond by-products face several challenges. Chemical and physical characteristics of almond by-products vary significantly depending on factors like almond cultivar, environmental conditions, and processing methods. This variability complicates standardization and limits broader application. Furthermore, phenolics and other bioactive compounds degrade due to heat, light, and oxygen exposure during processing and storage, reducing their efficacy in value-added products. Extracting high-purity bioactive compounds from complex plant matrices is technically challenging and costly. Minimizing the use of solvents and energy further complicates the process. Additionally, limited awareness of potential applications and regulatory hurdles in labeling and marketing products derived from almond by-products constrain industry growth.

The future economic importance of the almond industry must be based on three main pillars: the circular economy, the health and wellness industries, and global market growth. In this sense, an efficient use of almond by-products should align with sustainability objectives and should significantly reduce waste, providing additional revenue streams for almond producers. At the same time, expanding research into bioactive compounds could lead to new applications in disease prevention and management, increasing the economic value of almond-derived products. Additionally, growing consumer demand for sustainable, plant-based products provides a growing market for innovations in almond by-products, driving economic growth in the food, pharmaceutical, and cosmetic industries.

In conclusion, while challenges persist in standardizing and maximizing the utility of almond by-products, ongoing research and technological advancements hold promise for unlocking their full economic potential in a sustainable and environmentally friendly manner.

## Figures and Tables

**Table 1:** Chemical composition and nutritional content of almond hull.

Parameter	Content	References
**Routine Analyses**		
Dry matter (DM)	60.60–97.12 ^a^	[44,56,57,60]
Organic matter (OM)	86.87–93.90 ^a^	[56,58,66]
Volatile matter	71.20–75.73 ^a^	[30,66]
Moisture	0.65–11.30 ^a^	[30,66]
	6.95–9.01 (GH) ^a^	[61]
	6.01–9.80 (MH) ^a^	[61]
Crude protein (CP)	1.60–26.50 ^a^	[44,57,59,60,61]
Soluble crude protein (SCP)	1.40–57.00 ^a^	[56,69]
Sugar	15.90–34.30 ^a^	[44,57,58,59,60]
Ash	1.70–12.83 ^a^	[44,57,59,60,61,66,68]
Lipids	1.15–2.65 (GH) ^a^	[61]
	2.45–2.71 (MH) ^a^	[61]
Pectins	4.00 ^a^	[70]
Crude fiber (CF)	10.40–35.77 ^a^	[44,57,59,60,61]
Neutral detergent fiber (NDF)	18.00–61.98 ^a^	[56,57,58,59]
Acid detergent fiber (ADF)	12.60–34.60 ^a^	[56,57,58,59,62]
Soluble fiber	1.89–6.12 (GH) ^a^	[61]
	1.35–2.07 (MH) ^a^	[61]
Insoluble fiber	18.47–33.04 (GH) ^a^	[61]
	22.32–33.70 (MH) ^a^	[61]
Lignin	5.00–24.80 ^a^	[44,56,57,59,66]
Lignin/NDF	28.20–36.90 ^b^	[57]
Acid detergent lignin (ADL)	9.24–14.31 ^a^	[62,64,65,68]
Water Soluble carbohydrate (WSC)	12.63–14.13 ^a^	[68]
Nonfibrous carbohydrate (NFC)	5.04–70.96 ^a^	[56,62,64,65,68]
Nitrogen-free extract (NFE)	48.90–61.18 ^a^	[60,71,72]
Non-Structural Carbohydrates (NSC)	23.50–40.40 ^a^	[56]
Holocellulose	16.43 ^a^	[66]
Hemicellulose	6.00–12.86 ^a^	[44,65,66,67]
Cellulose	6.60–20.70 ^a^	[44,63,64,65,66]
Eter extract (EE)	0.40–8.20 ^a^	[57,58,59,60,65]
Ethanol-soluble carbohydrates	23.31–39.88 ^a^	[56]
N-free extract	61.18 ^a^	[71]
Starch	0.00–10.00 ^a^	[56,59]
Gross energy (GE)	15.10–19.70 ^c^	[59]
Carbon (C)	42.92–43.00 ^a^	[30,66]
Hydrogen (H)	5.70–5.80 ^a^	[30,66]
**Minerals**		
Calcium (Ca)	0.03–1.00 ^a^	[44,56,57,59,68]
	2.30–2.70 ^e^	[60]
Phosphorus (P)	0.00–2.00 ^a^	[56,57,59,60,68]
Magnesium (Mg)	0.07–0.40 ^a^	[56,57,59,69]
	0.09–0.12 ^e^	[60]
Potassium (K)	2.02–4.57 ^a^	[44,56,57,59,69]
	27.60–36.30 ^e^	[60]
Sodium (Na)	0.01–0.40 ^a^	[56,57,59,60]
Copper (Cu)	1.00–19.00 ^e^	[56,57,59,60,73]
Manganese (Mn)	5.00–69.00 ^e^	[56,59,60,73]
Iron (Fe)	0.08–0.71 ^d^	[56,59,60,73]
Aluminum (Al)	6.00–17.00 ^e^	[60]
Zinc (Zn)	6.00–63.00 ^e^	[56,59,60,73]
Nitrogen (N)	0.73–3.28 ^a^	[30,66]
Chlorine (Cl)	0.01–0.07 ^a^	[30,59,69]
Sulfur (S)	0.01–0.03 ^a^	[30,59,69]
Selenium (Se)	0.04–0.10 ^e^	[59,73]
Molybdenum (Mo)	0.00–12.1 ^e^	[69]
**Essential amino acids**		
Arginine	0.12–0.13 ^a^	[60]
Histidine	0.07 ^a^	[60]
Isoleucine	0.10–0.12 ^a^	[60]
Leucine	0.17–0.20 ^a^	[60]
Lysine	0.14–0.15 ^a^	[60]
Methionine	0.03–0.04 ^a^	[60]
Phenylalanine	0.12–0.13 ^a^	[60]
Threonine	0.11–0.13 ^a^	[60]
Tryptophan	<0.02 ^a^	[60]
Valine	0.15–0.17 ^a^	[60]

Units: ^a^—% DM. ^b^—ratio (%). ^c^—MJ/kg DM. ^d^—g/kg DM. ^e^—ppm. GH—green hull. MH—mature hull.

**Table 2:** Chemical composition and nutritional content of almond shell.

Parameter	Content	References
**Routine Analyses**		
Dry matter (DM)	84.80–93.20 ^b^	[59]
Moisture	3.30–11.20 ^a^	[30,80,81,82,85]
Volatile matter	73.00–81.20 ^a^	[30,78,81,82]
Crude protein (CP)	1.40–4.70 ^a^	[59,77]
Ash	0.55–8.70 ^a^	[76,77,78,79,80]
Crude fiber (CF)	51.80–62.00 ^a^	[59]
Neutral detergent fiber (NDF)	90.10 ^a^	[59]
Acid detergent fiber (ADF)	57.20–66.00 ^a^	[59]
Lignin	20.10–32.70 ^a^	[44,59,74,76,77,79]
Holocellulose	64.30 ^a^	[86]
Hemicellulose	19.70–35.20 ^a^	[44,52,74,75,76,77,78]
Cellulose	22.80–40.50 ^a^	[44,74,75,76,77,78]
Eter extract (EE)	0.20–1.10 ^a^	[59]
Gross energy (GE)	19.40 ^e^	[59]
Polysaccharides	56.10 ^a^	[79]
Carbon (C)	45.60–50.50 ^a^	[74,81,82,83]
Hydrogen (H)	5.40–6.60 ^a^	[74,81,82,83,84,85]
Oxygen (O)	37.97–45.94 ^a^	[74,81,82,83,84]
**Minerals**		
Calcium (Ca)	1.18–1.80 ^c^	[44,59,78,79]
Potassium (K)	4.30–12.30 ^c^	[44,59,78,79]
Phosphorus (P)	0.20–0.65 ^c^	[59,78,79]
Sodium (Na)	0.17–0.60 ^c^	[59,78,79]
Magnesium (Mg)	0.14–0.50 ^c^	[78,79]
Sulfur (S)	0.01–0.03 ^a^	[79,81,82,85]
Manganese (Mn)	0.01–0.03 ^c^	[78,79]
Zinc (Zn)	0.01 ^c^	[78,79]
Cooper (Cu)	3.20–10.00 ^d^	[78,79]
Iron (Fe)	0.04–1.64 ^c^	[59,78,79]
Molybdenum (Mo)	3.30 ^d^	[79]
Nitrogen (N)	0.17–0.44 ^a^	[74,81,82,85]
Boron (B)	0.01–0.02^c^	[78,79]
Cloride (Cl^-^)	0.02 ^a^	[85]
Chlorine (Cl)	0.05–0.04 ^a^	[30,59]

Units: ^a^—% DM. ^b^—% as fed. ^c^—g/kg DM. ^d^—mg/kg DM. ^e^—MJ/kg DM.

**Table 3:** Chemical composition and nutritional content of almond skin.

Parameter	Content	References
**Routine Analyses**		
Moisture	6.43–18.39 (BS) ^a^	[90,91]
Ash	1.63–5.20 (BS) ^a^	[88,91]
	4.80 (NS) ^a^	[88]
Protein	10.30 (NS) ^a^	[87,88]
	10.60–12.80 (BS) ^a^	[87,88,90,91]
Sugar	4.14–5.65 (BS) ^a^	[90,91]
Total Dietary Fiber (TDF)	47.50 (NS) ^a^	[87,89]
	45.10–60.25 (BS) ^a^	[87,88,89,90,91]
Soluble Dietary Fiber	2.70 (NS)–3.80 (BS) ^a^	[87,88]
Fat	10.30–22.20 (NS) ^a^	[87,88,89]
	9.50–24.20 (BS) ^a^	[87,88,89]
**Minerals**		
Manganese (Mn)	2.08 ^b^	[92]
Zinc (Zn)	2.96 ^b^	[92]
Cooper (Cu)	0.16 ^b^	[92]
Iron (Fe)	3.72 ^b^	[92]
Selenium (Se)	0.46 ^b^	[92]
**Vitamins**		
Vitamin E (α-Tocopherol)	13.00 (BS)–14.00 (NS) ^a^	[88]
**Fatty acids**		
Palmitic acid, 16:0	8.01–10.30 ^c^	[88,91,92]
Palmitoleic acid, 16:1	0.63–1.11 ^c^	[88,91,92]
Stearic acid, 18:1	1.37–2.39 ^c^	[88,91,92]
Oleic acid, 18:1	43.08–56.69 ^c^	[88,91,92]
Linoleic acid, 18:2	31.36–36.98 ^c^	[88,91,92]
α-Linolenic acid, 18:3	0.27–5.65 ^c^	[88,91,92]
MUFA	55.24–57.66 ^c^	[91]
PUFA	31.63–33.38 ^c^	[91]
SFA	10.71–11.39 ^c^	[91]

Units: ^a^—g/100 g. ^b^—mg/L in 1 g of skins. ^c^—mean percentages of fatty acid content in 100 g of almonds. BS—blanched almond skin. NS—natural almond skin. SFA—saturated fatty acids. MUFA—monounsaturated fatty acids. PUFA—polyunsaturated fatty acids.

**Table 4:** Content of total phenolics, flavonoids, *ortho*-diphenols, condensed tannins, and antioxidant capacity found in almond by-products.

Almond By-Product	Total Phenolics	Flavonoids	*Ortho*-Diphenols	Condensed Tannins	Antioxidant Capacity	References
Hull	18,307.26–22,593.33 ^i^	–	–	–	29,250.00–44,424.00 ^p^	[36]
	103.44–184.53 ^d^	–	–	–	671.78–1159.83 ^l^	[61]
	3.08–210.49 ^d^	0.87–120.04 ^a^	–	0.09–123.54 ^a^	23.43–1938.07 ^j^	[121]
	7.90–32.66 ^d^	4.28–29.05 ^u^	8.28–24.53 ^d^	–	0.07–0.28 ^x^	[32]
	91.76–138.90 ^d^	36.99–125.35 ^a^	107.34–131.34 ^d^	–	0.85–1.54 ^x^	[120]
	32.00–35.70 ^h^	–	–	23.20–28.40 ^h^	–	[62,68]
	304.78–859.07 ^d^	70.48–284.61 ^u^	–	–	169.85–376.30 ^y^	[122]
	35.90–166.70 ^d^	–	–	–	29.70–98.70 ^q^	[123]
	71.00 ^b^	–	–	–	–	[35]
	71.10 ^u^	–	–	–	41.10 ^o^	[124]
Shell	6.59 ^d^	1.42 ^a^	–	–	4.21–6.20 ^k^	[119]
	188.60 ^d^	99.40 ^u^	–	34.60 ^u^	646.00 ^k^	[79]
	3.55–8.62 ^d^	1.74–6.05 ^a^	3.43–9.95 ^d^	–	0.03–0.10 ^x^	[120]
	18.40–62.70 ^d^	–	–	–	29.3–63.50 ^q^	[123]
	13.73–19.76 ^c^	–	–	–	27.90–82.70 ^y^	[125]
Natural Skin	703.03 ^e^	–	–	–	6034.43–16,259.40 ^m^	[116]
	3471.10 ^e^	–	–	–	0.20 ^y^	[89]
	27.60 ^d^	–	–	–	210.00 ^l^	[126]
Blanched Skin	1.72–7.07 ^c^	0.52–1.20 ^v^	–	–	–	[91]
	–	–	–	–	80.17 ^n^	[92]
	13.44–34.71 ^d^	11.14–34.43 ^u^	10.65–26.59 ^d^	–	0.06–0.18 ^x^	[32]
	88.00 ^b^	–	–	–	–	[35]
	7.62–25.17 ^d^	4.45–13.66 ^a^	6.95–23.32 ^d^	–	0.04–0.30 ^x^	[120]
	1110.00–1773.00 (ND) ^f^	–	–	–	40.40 ^l^ (ND)	[39]
	253.60–857.00 ^f^	–	–	–	59.20–90.40 ^l^	[39]
	313.76 ^e^	–	–	–	2925.15–7363.06 ^m^	[116]
	165.00–370.00 ^f^		–		–	[115]
	278.90 ^e^	–	–	–	6.50 ^y^	[89]
	242.00–413.00 ^f^	–	–	–	0.40–0.50 ^g^	[114]
	88.00 ^b^					[35]
	87.80 ^u^	–	–	–	52.90 ^o^	[124]
Roasted Skin	–	–	–	–	0.80–1.08 ^g^	[127]
	18.50 ^d^				119.00 ^l^	[126]
Skin (B + D)	–	–	–	–	0.40–0.58 ^g^	[127]
Skin (B + FD)	–	–	–	–	0.33–0.45 ^g^	[127]
Blanching Water	392.16–505.95 ^r^	292.78–467.78 ^t^	224.21–318.07 ^r^	–	1.98– 3.64 ^w^	[32]
	510.00–917.00 ^s^	–	–	–	17.40 ^l^	[39]
	0.56–3.36 ^c^	0.18–0.77 ^v^	–		–	[91]
	73.86 ^e^	–	–	–	575.08–1049.95 ^m^	[116]
	90.28 ^d^	–	–	–	132.82 ^y^	[128]
	33.30 ^e^					[89]
	50.30–153.90 ^e^	–	–	–	–	[33]

Legend: ^a^—mg CATE/g. ^b^—mg QE/g. ^c^—g GAE/100 g. ^d^—mg GAE/g. ^e^ mg GAE/100 g FW. ^f^—µg/g. ^g^—ORAC values (mmol TE/g). ^h^—g/kg. ^i^—mg GAE/100 mL. ^j^—µM TE/g. ^k^—mg TE/g. ^l^—µmol TE/g. ^m^—µmol TE/100 g FW. ^n^—µM TE/100 g. ^o^—ABTS values (mg TE/mL). ^p^—µmol TE/100 mL. ^q^—% hydrogen peroxide-scavenging capacities. ^r^—mg GAE/L. ^s^– mg/L. ^t^—mg CATE/L. ^u^—mg CATE/g. ^v^—g RE/100 g. ^w^—mmol TE/L. ^x^—mmol TE/g. ^y^—% of inhibition (EC50). GAE—gallic acid equivalents. CATE—catechin equivalents. QE—quercetin equivalents. TE—Trolox equivalents. RE—Rutin equivalents. ND—not dried. B + D—blanching + drying. B + FD—blanching + freeze-dried. FW—fresh weight. –not found.

**Table 5:** Main bioactivities of almond by-products reported in the literature.

Almond By-Products	Bioativities	Main Compounds Responsible	References
Hull	Antioxidant	Polyphenols (such as chlorogenic acid)	[11,32,116,121,123,129,171]
	Antimicrobial	Polyphenols (such as naringenin, catechin epicat-echin, protocatechuic acid, isorhamnetin)	[11,36,38,183,184]
	Antitumor/Anticancer	Polyphenols, acid-soluble polysaccharides, triterpenoids acidsand UFAs	[17,154,178,199,200,201,202,203]
Shell	Antioxidant	Phenolic compounds	[123]
Skin (Natural and Blanched)	Antioxidant	Polyphenols (mainly flavonols and proanthocyanidins)	[11,32,38,39,50,113,114,137,172,175,176,203]
	Antitumor/Anticancer	Polyphenols, acid-solubles polysacharides, triterpenoids acis and UFAs	[11,178,199,201,202,203]
	Antimicrobial	Polyphenols (such as naringenin, catechin epicatechin, protocatechuic acid, isorhamnetin)	[11,32,38,116,152,158,181,182]
	Photoprotective	Polyphenols	[128,156]
	Anti-inflammatory	Polyphenols, UFAs, protein hidrolysates	[11,153,185,187]
	Prebiotic	Dietary Fibers, XOS, polysaccharides, and hemicellulose	[11,155,178,188,198]
Blanching Water	Antioxidant	Polyphenols	[11,32,137]
	Antimicrobial	Polyphenols	[176,178]
	Anti-inflammatory	Polyphenols	[186]
	Photoprotective	Polyphenols	[128,156]

## Data Availability

No new data were created or analyzed in this study. Data sharing is not applicable to this article.

## Supplementary Materials

The following supporting information can be downloaded at: https://www.mdpi.com/article/10.3390/foods14061042/s1, Table S1: Main bioactive compounds in almond by-products.
