# Supplementary material for: Almond By-Products: A Comprehensive Review of Composition, Bioactivities, and Influencing Factors
Source: Foods. 2025 Mar 19;14(6):1042. doi: 10.3390/foods14061042 (PMC11941897; doi:10.3390/foods14061042)
Supplement: Supplementary file 1 [file foods-14-01042-s001.zip › foods-3492411-supplementary.pdf]

Table S1. Main bioactive compounds in almond by-products.

PHENOLIC COMPOUNDS ǀ *Non-Flavonoids*			
**Almond By-Product**	**Compound**	**Content**	**References**
Phenolic Acids – **Benzoic Acids (BAs) ǀ Hridroxybenzoic Acids (HBAs)**			
Skin	*p*-hydroxybenzoic acid (*p*-HBA)	+	[112,111,118]
		17.50 [d]	[33]
		1.31–7.18 [a]	[32,114,115,127]
		887.85 (B)–6,401.20 (N) [b]	[88]
		63.40–136.00 [b]	[117]
		127.17 (B)–477.56 (N) [e]	[116]
		3.60-32.80 [h] (ND)	[39]
		1.50–65.90 [h]	[39]
		0.01–0.09 [c]	[91]
	Vanillic acid	+	[118,112]
		18.20 [d]	[33]
		7.65–19.20 [a]	[115,114,127]
		861.44 (B)–5,805.23 (N) [b]	[88]
		425.15(B)–3,436.60 (N) [e]	[116]
		10.80–19.90 (ND) [h]	[39]
		1.50–30.70 [h]	[39]
		9.58–16.50 [c]	[91]
	Protocatechuic acid	+	[112,111,118]
		17.60 [d]	[33]
		2.22–32.00 [a]	[32,114,115,127]
		426.90 (B)–1,541.67 (N) [b]	[88]
		285.12 (B)–2,319.70 (N) [e]	[116]
		3.20-92.40 [h] (ND)	[39]
		2.90–201.00 [h]	[39]
		9.37–16.69 [c]	[91]
Hull	*p*-hydroxybenzoic acid (*p*-HBA)	2.87–3.66 [a]	[32]
	Vanillic acid	0.49–2.32 [a]	[32]
	Protocatechuic acid	+	[36,109]

Table S1. Main bioactive compounds in almond by-products (Cont.).

**PHENOLIC COMPOUNDS ǀ *Non-Flavonoids***			
**Almond By-Product**	**Compound**	**Content**	**References**
***Phenolic Acids* – Benzoic Acids (BAs) ǀ Hridroxybenzoic Acids (HBAs)**			
**Hull**	Protocatechuic acid	2.22–3.66 [a]	[32]
	Prenylated benzoic acid	+	[109]
**Blanching Water**	*p*-hydroxybenzoic acid (*p*-HBA)	3.56–542.37 [a]	[32,128]
		82.50 [d]	[33]
		223.26 [b]	[88]
		97.81 [e]	[116]
		2.94–4.91[g]	[39]
		0.44–1.31 [c]	[91]
	Vanillic acid	63.54 [a]	[128]
		72.60 [d]	[33]
		18.78 [b]	[88]
		1.50–30.70 [h]	[39]
		531.22 [e]	[116]
		1.98–11.67 [c]	[91]
	Protocatechuic acid	15.71–73.19 [a]	[32,128]
		70.20 [d]	[33]
		36.55 [b]	[88]
		453.86 [e]	[116]
		5.68–5.89 [g]	[39]
		1.38–3.03 [c]	[91]
***Phenolic Acids* – Benzoic Acid Aldehydes (Benzaldehydes) ǀ Hydroxybenzaldehydes**			
**Almond Skin**	Protocatechuic aldehyde	5.45–20.10 [a]	[114,115]
***Phenolic Acids* – Cinnamic Acids ǀ Hydroxycinnamic Acids**			
**Skin**	Chlorogenic acid	1.76–13.77 [a]	[114,115,127]
		+	[112]
		9,571.26 (N)–1,190.84 (B) [b]	[88]
		62.21(B)–5,221.13 (N) [e]	[116]
		4.38–8.66 [c]	[91]

Table S1. Main bioactive compounds in almond by-products (Cont.).

**PHENOLIC COMPOUNDS** ǀ ***Non-Flavonoids***			
**Almond By-Product**	**Compound**	**Content**	**References**
Phenolic Acids **– Cinnamic Acids ǀ Hydroxycinnamic Acids**			
**Skin**	*trans-p*-coumaric acid	0.31–4.55 [a]	[32,35,114,115,127]
		5.80–8.10 [h]	[39]
		+	[112]
		67.30 (B)–367.71(N) [b]	[88]
		31.87 (B)–228.63 (N) [e]	[116]
		0.01–0.55 [c]	[91]
	Caffeic acid	Traces	[35]
	Sinapic acid	9.51 [a]	[35]
	Ferulic acid	2.19 [a]	[35]
**Hull**	Chlorogenic acid	42.52 [c]	[110]
		12.51–122.03 [c]	[36]
		1.64–111.05 [i]	[36]
		4.80–19.62 [a]	[32]
	Cryptochlorogenic acid	7.90 [c]	[110]
		5.85–10.34 [c]	[36]
		5.15–9.36 [i]	[36]
	Neochlorogenic acid	3.04 [c]	[110]
		1.75–13.28 [c]	[36]
		1.57–11.68 [i]	[36]
	trans-p-coumaric acid	1.34–4.32 [a]	[32,35]
	Caffeic acid	Traces	[35]
	Sinapic acid	9.92 [a]	[35]
	Ferulic acid	2.71 [a]	[35]
**Blanching Water**	*trans-p*-coumaric acid	15.29 [a]	[128]
		0.07 [c]	[91]
		3.99 [b]	[88]
		13.90 [e]	[116]
	Chlorogenic acid	18.05–84.32 [a]	[32,123]

Table S1. Main bioactive compounds in almond by-products (Cont.).

**PHENOLIC COMPOUNDS ǀ** ***Non-Flavonoids***			
**Almond By-Product**	**Compound**	**Content**	**References**
Phenolic Acids **– Cinnamic Acids ǀ Hydroxycinnamic Acids**			
**Blanching Water**	Chlorogenic acid	0.01–0.03 [c]	[91]
		42.49 [b]	[88]
		4.66 [e]	[116]
Stilbenes			
**Skin**	Polydatin	0.15–0.22 [b]	[147]
**Blanching Water**	Polydatin	6.33–8.43 [b]	[147]
	Piceatannol + oxyresveratrol [A]	0.91–2.55 [b]	[147]
**PHENOLIC COMPOUNDS ǀ** ***Flavonoids***			
Flavanols **– Favan-3-ols** (monomeric and oligomeric forms)			
**Skin**	(+)-Catechin	+	[111,112,118]
		35.70 [d]	[33]
		7.30–90.10 [a]	[113–115,127]
		5,057.07(B)–15,569.00 (N) [b]	[88]
		579.00–1,380.00 [b]	[117]
		183.67 (B)–588.49 (N) [e]	[116]
		126.00–1,085.00 [h] (ND)	[39]
		15.60–1,216.00 [h]	[39]
		9.69–38.26 [c]	[91]
	(−)-Epicatechin	33.90 [d]	[33]
		1.3–36.60 [a]	[32,113–115,127]
		+	[111,112]
		195.00–399.00 [b]	[117]
		1,589.58 (B)–10,955.64(N) [b]	[88]
		52.35 (B)–233.80 (N) [e]	[116]
		20.60–1,393.00 [h] (ND)	[39]
		29.40–294.00 [h]	[39]
		3.53–10.60 [c]	[91]

Table S1. Main bioactive compounds in almond by-products (Cont.).

**PHENOLIC COMPOUNDS** ǀ ***Flavonoids***			
**Almond By-Product**	**Compound**	**Content**	**References**
***Flavanols* – Favan-3-ols** (monomeric and oligomeric forms)			
**Skin**	Procyanidin $B_3 + B_1$	11.08–23.80 [a]	[114,115]
	Procyanidin B_2	2.34–16.10 [a]	[114,115]
	Procyanidin B_5	2.29–8.57 [a]	[114,115]
	Procyanidin B_7	3.72–13.90 [a]	[114,115]
	Procyanidin C_1	3.45–15.30 [a]	[114,115]
	A-type procyanidin dimer	0.70–7.29 [a]	[114]
	Unknown dimer A [(epi)catechin →A→(epi)catechin]	1.16–4.93 [a]	[115]
**Hull**	(+)-Catechin	+	[36,109]
**Blanching Water**	(+)-Catechin	693.41 [a]	[128]
		55.60 [d]	[33]
		231.38 [b]	[88]
		47.76 [e] (N)	[116]
		408.00–847.00 [g]	[39]
		0.01–3.95 [c]	[91]
	(−)-Epicatechin	376.09 [a]	[128]
		62.10 [d]	[33]
		127.31 [b]	[88]
		20.82 [e]	[116]
		30.90–63.90 [g]	[39]
		0.01 [c]	[91]
Flavonols ǀ **Flavonol glycosides**			
**Skin**	Quercetin-3-*O*-galactoside	41.40 [d]	[33]
		140.19 (B)–1339.65 (N) [b]	[88]
		60.62 (B)–120.57 (N) [e]	[116]
		0.07–10.10 [c]	[91]

Table S1. Main bioactive compounds in almond by-products (Cont.).

**PHENOLIC COMPOUNDS** ǀ ***Flavonoids***			
**Almond By-Product**	**Compound**	**Content**	**References**
Flavonols ǀ **Flavonol glycosides**			
Skin	Quercetin-3-*O*-glucoside	24.50 [d]	[33]
		0.00–2.41 [a]	[114,115,127]
		+	[111,112]
		60.51 (B)–896.45 (N) [b]	[88]
		26.00–86.00 [b]	[117]
		166.93 (B)–400.32 (N) [e]	[116]
		0.02–0.06 [c]	[91]
	Quercetin-3-*O*-rutinoside (Rutin)	43.70 [d]	[33]
		+	[111]
		550.70 (B)–3197.65 (N) [b]	[88]
		1.20–35.00 [b]	[117]
		960.15 (B)–1,355.27 (N) [e]	[116]
		0.04–0.12 [c]	[91]
	Quercetin-3-*O*-rhamnoside (Quercitrin)	+	[112]
		1.22–19.63 [c]	[91]
	Kaempferol-3-*O*-rutinoside	+	[34,111,150]
		40.40 [d]	[33]
		1.00–238.70 [a]	[113,114,115,127]
		12.30–208.00 [b]	[117]
		4,076.25 (B)–22,949.27 (N) [b]	[88]
		10,829.87 (B)–24,166.19 (N) [e]	[116]
		31.60–69.10 [h] (ND)	[39]
		7.80–49.70 [h]	[39]
		0.40–1.50 [c]	[91]
	Kaempferol-3-*O*-galactoside	36.40 [d]	[33]
		+	[111]
	Kaempferol rutinoside	+	[144,145]

Table S1. Main bioactive compounds in almond by-products (Cont.).

Almond By-Product	Compound	Content	References
**PHENOLIC COMPOUNDS | *Flavonoids***			
Flavonols | **Flavonol glycosides**			
Skin	Kaempferol glucoside	+	[144,145]
	Kaempferol-3-*O*-glucoside	39.00 [d]	[33]
		0.00–14.20 [a]	[32,114,115,127]
		+	[111]
		1,124.97 (B)–39,012.26 (N) [b]	[88]
		18.50–222.00 [b]	[117]
		4,080.93 (B)–23,070.37 (N) [e]	[116]
		4.4 [h] (ND)	[39]
		0.70–33.30 [h]	[39]
		1.00–26.58 [c]	[91]
	Isorhamnetin-3-*O*-glucoside	+	[34,111]
		0.87–32.50 [a]	[32,113,114,115,127]
		895.46 (B)–16,940.91 (N) [b]	[88]
		126.00–1,964.00 [b]	[117]
		461.25 (B)–2,370.82 (N) [e]	[116]
		63.07–122.94 [c]	[91]
	Isorhamnetin-3-*O*-galactoside	35.00 [d]	[33]
	Isorhamnetin glucoside	+	[144,145]
	Isorhamnetin rutinoside	+	[144,145]
	Isorhamnetin-3-*O*-rutinoside (and Isorhamnetin-3-*O*-glucoside)	28.50 [d]	[33]
	Isorhamnetin-3-*O*-rutinoside	5.34–756.50 [a]	[32,113,114,115,127]
		+	[111,112]
		5,379.92 (B)–54,901.07 (N) [b]	[88]
		117.00–1,745.00 [b]	[117]
		210.79 (B)–1,812.86 (N) [e]	[116]
		20.40–105.00 [h]	[39]

Table S1. Main bioactive compounds in almond by-products (Cont.).

**PHENOLIC COMPOUNDS** ǀ ***Flavonoids***			
**Almond By-Product**	**Compound**	**Content**	**References**
Flavonols ǀ **Flavonol glycosides**			
**Skin**	Isorhamnetin-3-*O*-rutinoside	48.30–110.00 [h] (ND)	[39]
		0.44 [c]	[91]
**Hull**	Kaempferol-3-*O*-glucoside	0.34–1.19 [a]	[32]
	Kaempferol-3-*O*-rutinoside	+	[34]
		0.37–1.73 [a]	[32]
	Isorhamnetin-3-*O*-glucoside	+	[34]
		0.37–0.92 [a]	[32]
	Isorhamnetin-3-*O*-rutinoside	1.84–7.96 [a]	[32]
		20.82 [b]	[88]
		333.12 [e]	[116]
**Blanching Water**	Quercetin-3-*O*-glucoside	7.13 [a]	[128]
		75.50 [d]	[33]
		2.42 [b]	[88]
		0.06–0.13 [c]	[91]
	Quercetin-3-*O*-rutinoside (Rutin)	79.43 [a]	[128]
		56.30 [d]	[33]
		25.18 [b]	[88]
		11.32 [e]	[116]
		0.05–0.19 [c]	[91]
	Quercetin-3-*O*-galactoside	58.60 [d]	[33]
		34.47 [e]	[116]
		0.02–0.28 [c]	[91]
	Quercetin-3-*O*-rhamnoside (Quercitrin)	0.12–1.82 [c]	[91]
	Kaempferol-3-*O*-rutinoside	803.54 [a]	[128]
		52.60 [d]	[33]
		276.09 [b]	[88]

Table S1. Main bioactive compounds in almond by-products (Cont.).

**PHENOLIC COMPOUNDS** ∣ *Flavonoids*			
**Almond By-Product**	**Compound**	**Content**	**References**
Flavonols ∣ **Flavonol glycosides**			
Blanching Water	Kaempferol-3-*O*-rutinoside	6,247.21 [e]	[116]
		8.10–11.50 [g]	[39]
		0.02–0.07 [c]	[91]
	Kaempferol-3-*O*-glucoside	33.73 [a]	[128]
		44.50 [d]	[33]
		8.78 [b]	[88]
		2,080.24 [e]	[116]
		1.46 [g]	[39]
		15.72–20.68 [a]	[32]
		0.03–0.26 [c]	[91]
	Kaempferol-3-*O*-galactoside	43.50 [d]	[33]
	Isorhamnetin-3-*O*-rutinoside (and Isorhamnetin-3-*O*-glucoside)	68.60 [d]	[33]
	Isorhamnetin-3-*O*-galactoside	61.70 [d]	[33]
	Isorhamnetin-3-*O*-glucoside	123.59 [a]	[128]
		54.28 [b]	[88]
		56.57 [e]	[116]
		3.64 [g]	[39]
		27.91–41.71 [c]	[91]
	Isorhamnetin-3-O-rutinoside	42.12–80.33 [a]	[32,128]
		13.30–18.60 [g]	[39]
Flavonols ∣ **Flavonol aglycones**			
Skin	Kaempferol	100.00 [d]	[33]
		1.71–12.10 [a]	[114,115,127]
		+	[111,112]
		291.00 (B)–1,249.97 [b] (N)	[88]
		9.50–48.90 [b]	[117]
		3,982.85 (B)–23,845.52(N) [e]	[116]

Table S1. Main bioactive compounds in almond by-products (Cont.).

**PHENOLIC COMPOUNDS** ǀ *Flavonoids*			
**Almond By-Product**	**Compound**	**Content**	**References**
Flavonols ǀ **Flavonol aglycones**			
**Skin**	Kaempferol	9.10–14.90 [h] (ND)	[39]
		0.30–7.5 [h]	[39]
		0.11 c	[89]
	Quercitrin	+	[34]
	Quercetin	+	[34,111,112]
		100.00 [d]	[33]
		1.02–4.89 [a]	[114,115,127]
		147.43 (B)–214.30 (N) [b]	[88]
		13.90–31.60 [b]	[117]
		802.16 (B)–2,547.06 (N)[e]	[116]
		0.01 [c]	[91]
	3′-*O*-methylquercetin 3-*O*-*β*-D-glucopyranoside (Quercetin as aglycon)	+	[118]
	3′-*O*-methylquercetin 3-*O*-*β*-D-galactopyranoside (Quercetin as aglycon)	+	[118]
	3′-*O*-methylquercetin 3-*O*-*α*-L-rhamnopyranosyl-(1→6)-*β*-D-glucopyranoside (Quercetin as aglycon)	+	[118]
	Kaempferol 3-*O*-*α*-L-rhamnopyranosyl-(1→6)-β-D-glucopyranoside	+	[118]
	Isorhamnetin	47.20 [d]	[33]
		+	[34,111,112]
		1.40–16.00 [a]	[32,114,115,127]
		492.29 (B)–4,551.85 (N) [b]	[88]
		50.10–158.00 [b]	[117]

Table S1. Main bioactive compounds in almond by-products (Cont.).

PHENOLIC COMPOUNDS ǀ *Flavonoids*			
**Almond By-Product**	**Compound**	**Content**	**References**
Flavonols ǀ **Flavonol aglycones**			
**Skin**	Isorhamnetin	68.65 [e] (N)	[116]
		9.70–38.60 [h] (ND)	[39]
		6.70–36.10 [h]	[39]
		3.70–13.44 [c]	[91]
	Morin	+	[34]
**Hull**	Quercetin	+	[34]
	Isorhamnetin	+	[34]
		0.48–1.31 [a]	[32]
	Quercitrin	+	[34]
	Morin	+	[34]
**Blanching Water**	Kaempferol	2,392.44 [e]	[116]
		0.05–0.08 [c]	[91]
	Quercetin	31.04 [e]	[116]
		0.01–2.10 [c]	[91]
	Isorhamnetin	50.60 [d]	[33]
		0.04–0.27 [g]	[39]
		0.01–0.04 [c]	[91]
Flavanones ǀ *Flavanone* **aglycones**			
**Skin**	Naringenin	34.10 [d]	[33]
		2.83–12.10 [a]	[114,115]
		+	[111]
		1381.77 (B)–20,634.38 [a] (N)	[88]
		17.50–184.00 [b]	[117]
		21,628.65 (B)–25,122.26 (N) [e]	[116]
		2.90 [h] (ND)	[39]
		1.70–2.90 [h]	[39]
		1.66–3.94 [c]	[91]

Table S1. Main bioactive compounds in almond by-products (Cont.).

**PHENOLIC COMPOUNDS** ǀ *Flavonoids*			
**Almond By-Product**	**Compound**	**Content**	**References**
*Flavanones*ǀ *Flavanone* **aglycones**			
**Skin**	Eriodictyol	48.70 [d]	[33]
		2.34–7.76 [a]	[114,115]
		+	[111]
		41.74 (B)–470.09 (N) [b]	[88]
		249.08 (B)–1,437.32 (N) [e]	[116]
		0.01 [c]	[91]
**Blanching Water**	Naringenin	37.80 [d]	[33]
		14.70–53.72 [a]	[88,128]
		+	[113]
		0.12 [g]	[39]
		0.01–0.04 [c]	[91]
	Eriodictyol	51.30 [d]	[33]
		4.69 [b]	[88]
		216.94 [e]	[116]
		0.01–0.09 [c]	[91]
		16.27 [a]	[128]
Flavanones ǀ **Flavanone glycosides**			
**Skin**	Naringenin-7-*O*-glucoside	16.10 [d]	[33]
		1.50–25.90 [a]	[32,114,115,127]
		+	[111,112,113]
		961.61 (B)–14,288.10 (N) [b]	[88]
		72.40–166.00 [b]	[117]
		10.50–17.40 [h] (ND)	[39]
		2.60–20.80 [h]	[39]
		9.41–20.59 [c]	[91]
	Eriodictyol-7-*O*-glucoside	0.81–2.65 [a]	[32,114,115,127]
		+	[112]
		77.88 (B)–3,382.47 (N)[b]	[88]

Table S1. Main bioactive compounds in almond by-products (Cont.).

Almond By-Product	Compound	Content	References
**PHENOLIC COMPOUNDS ǀ *Flavonoids***			
***Flavanones* ǀ Flavanone glycosides**			
**Skin**	Eriodictyol-7-*O*-glucoside	1,309.15 (B)–3,696.85 (N) [e]	[116]
		1.08–3.20 [c]	[91]
	Naringenin 7-*O*-β-D-glucopyranoside (Prunin)	+	[118]
**Hull**	Naringenin-7-*O*-glucoside	19.62–105.56 [a]	[32]
**Blanching Water**	Naringenin-7-*O*-glucoside	815.38 [a]	[128]
		73.40 [d]	[33]
		286.92 [b]	[88]
		0.31–0.49 [g]	[39]
		0.11–0.97 [c]	[91]
	Eriodictyol-7-*O*-glucoside	4.85–34.35 [a]	[32,123]
	Eriodictyol-7-*O*-glucoside	10.96 [b]	[88]
		124.67 [e]	[116]
		0.01 [c]	[91]
Flavanonols			
**Skin**	Quercetin dihydrate (Dihydroquercetin or Taxifolin)	0.80–2.80 [h]	[39]
		0.00–10.30 [a]	[115]
		+	[114]
**TERPENOIDS**			
Sterols			
**Hull**	Stigmasterol	18.90 [c]	[110]
	β-sitosterol	16.00 [c]	[110]
Triterpenoids			
**Hull**	Betulinic acid	57.98-160.94 [c]	[36]
		38.39-75.29 [i]	[36]
	Oleanolic acid	133.82-285.71 [c]	[36]
		60.26-204.88 [i]	[36]

Table S1. Main bioactive compounds in almond by-products (Cont.).

**TERPENOIDS**			
**Almond By-Product**	**Compound**	**Content**	**References**
Triterpenoids			
**Hull**	Ursolic acid	+	[109]
		68.97-162.83 [c]	[36]
		41.69-75.41 [i]	[36]

Legend: [+] – present. [a] – μg/g of extract. [b] – μg/100 g. [c] – mg/100 g. [d] – % percent distribution from almonds. [e] – μg/100 g fresh weight. [f] – mg/g of extract. [g] – mg/L of blanching water. [h] – μg/g dry matter. [i] mg/mL of extract. [A] – Quantitated as piceatannol equivalents. B – Blanched almond skin. N – Natural almond skin. ND – not dried.
